# Metagenome Assembled Genome of a Novel Verrucomicrobial Methanotroph From Pantelleria Island

**DOI:** 10.3389/fmicb.2021.666929

**Published:** 2021-05-19

**Authors:** Nunzia Picone, Pieter Blom, Carmen Hogendoorn, Jeroen Frank, Theo van Alen, Arjan Pol, Antonina L. Gagliano, Mike S. M. Jetten, Walter D’Alessandro, Paola Quatrini, Huub J. M. Op den Camp

**Affiliations:** ^1^Department of Microbiology, Institute for Water and Wetland Research (IWWR), Radboud University, Nijmegen, Netherlands; ^2^Istituto Nazionale di Geofisica e Vulcanologia, Sezione di Palermo, Palermo, Italy; ^3^Department of Biological, Chemical and Pharmaceutical Sciences and Technologies (STEBICEF), University of Palermo, Palermo, Italy

**Keywords:** Verrucomicrobia, acidophilic, methanotroph, *Ca*. Methylacidithermus pantelleriae, volcanic soil

## Abstract

Verrucomicrobial methanotrophs are a group of aerobic bacteria isolated from volcanic environments. They are acidophiles, characterized by the presence of a particulate methane monooxygenase (pMMO) and a XoxF-type methanol dehydrogenase (MDH). Metagenomic analysis of DNA extracted from the soil of Favara Grande, a geothermal area on Pantelleria Island, Italy, revealed the presence of two verrucomicrobial Metagenome Assembled Genomes (MAGs). One of these MAGs did not phylogenetically classify within any existing genus. After extensive analysis of the MAG, we propose the name of “*Candidatus* Methylacidithermus pantelleriae” PQ17 gen. nov. sp. nov. The MAG consisted of 2,466,655 bp, 71 contigs and 3,127 predicted coding sequences. Completeness was found at 98.6% and contamination at 1.3%. Genes encoding the pMMO and XoxF-MDH were identified. Inorganic carbon fixation might use the Calvin-Benson-Bassham cycle since all genes were identified. The serine and ribulose monophosphate pathways were incomplete. The detoxification of formaldehyde could follow the tetrahydrofolate pathway. Furthermore, “*Ca.* Methylacidithermus pantelleriae” might be capable of nitric oxide reduction but genes for dissimilatory nitrate reduction and nitrogen fixation were not identified. Unlike other verrucomicrobial methanotrophs, genes encoding for enzymes involved in hydrogen oxidation could not be found. In conclusion, the discovery of this new MAG expands the diversity and metabolism of verrucomicrobial methanotrophs.

## Introduction

Verrucomicrobial methanotrophs are a group of aerobic bacteria usually found in the acidic soil of geothermal active regions (Dunfield et al., 2007; Pol et al., 2007; Islam et al., 2008; Sharp et al., 2014; van Teeseling et al., 2014). Their genomes all encode one or up to three particulate methane monooxygenase enzymes (pMMO) for the conversion of methane to methanol and a XoxF-type methanol dehydrogenase (MDH) to transform methanol to formate. The peculiarity of their XoxF-MDH is the strict dependence on rare earth elements (REEs), which are present in the active site together with the pyrroloquinoline quinone (PQQ) cofactors (Pol et al., 2014). Formate is ultimately converted to CO_2_ by a formate dehydrogenase (Picone and Op den Camp, 2019).

Inorganic carbon is fixed autotrophically using the Calvin-Benson-Bassham (CBB) cycle rather than the serine- or ribulose monophosphate (RuMP) pathways used by proteobacterial methanotrophs (Khadem et al., 2011; van Teeseling et al., 2014). Two verrucomicrobial methanotrophs were shown to be able to grow in the absence of methane when supplied with a mixture of carbon dioxide and hydrogen (Mohammadi et al., 2017a; Schmitz et al., 2020). Moreover, they are capable of nitrogen fixation and partial denitrification (Khadem et al., 2010; Mohammadi et al., 2017b). The current classification divides verrucomicrobial methanotrophs into two genera: *Methylacidimicrobium*, generally mesophilic and extremely acidophilic and the thermophilic but less acidophilic *Methylacidiphilum* (Dunfield et al., 2007; Pol et al., 2007; Islam et al., 2008; Sharp et al., 2014; van Teeseling et al., 2014; Picone et al., 2021).

Verrucomicrobial methanotrophs were detected in different geothermal ecosystems, including the Favara Grande, a volcanic area on Pantelleria Island, Italy. In particular, *pmo*-containing bacteria closely related to *Methylacidiphilum fumariolicum* SolV, were detected in site FAV2 (Gagliano et al., 2014). This site was characterized by pH values of 4–4.5 and temperature ranging from 60°C in the top layer of the soil, to 92°C at 50 cm depth. Ammonia was scarce, whereas high emissions of carbon dioxide (CO_2_), hydrogen (H_2_), and methane (CH_4_) were recorded (Gagliano et al., 2016). A recent metagenomic analysis of site FAV2 revealed a methanotrophic community composed of Proteobacteria and Verrucomicrobia (Picone et al., 2020), supporting the findings of Gagliano et al. (2014). Two Metagenome Assembled Genomes (MAGs) that belonged to the phylum Verrucomicrobia were retrieved. One of these MAGs (MAG5) was a novel *Methylacidimicrobium* species (Picone et al., 2021). MAG9, instead, did not classify as *Methylacidiphilum* or *Methylacidimicrobium*, indicating that it may represent a novel genus.

In this study we determine the phylogeny of this new verrucomicrobial methanotroph species and analyze its genome to predict the metabolic potential.

## Materials and Methods

### Sampling

Samples were collected in June 2017 in the area of Favara Grande on Pantelleria Island, Italy (23°21′77′′N; 40°73′160′′E). Soil samples were taken using a core sampler (diameter 1.5 cm) and deposited in sterile 50 mL tubes. Tubes were stored at 4°C till further analysis. For a more extensive description of the sampling site (see Picone et al., 2020).

### DNA Extraction and Sequencing

DNA was extracted from soil samples using two different methods: Fast DNA Spin kit for soil (MP Biomedicals, Santa Ana, California), according to manufacturer’s instructions, and CTAB DNA extraction (Allen et al., 2006). Cell lysis within the CTAB method was performed by incubating 250 mg of soil with 675 μl of CTAB buffer (100 mM Tris, 100 mM EDTA, 100 mM Na2HPO4, 1.5 M NaCl and 1% CTAB), 50 μl of lysozyme (10 mg/ml, 66,200 U/mg), and 30 μl of Rnase A (10 mg/ml) for 30 min at 37°C. Fifty microliter of Proteinase K (10 mg/ml, 20 U/mg) was added to the sample and incubated for 30 min at 37°C. Next, 150 μl of 10% SDS was added and the mixture was incubated for 2 h at 65°C. DNA was extracted by adding 1 volume of phenol/chloroform/isoamylalcohol (25:24:1) and incubating the sample for 20 min at 65°C. Supernatant was treated with 1 volume of chloroform/isoamylalcohol (24:1) and centrifuged for 10 min at 20,000 × *g*. Next, 0.6 volume of isopropanol was added to the aqueous phase and DNA was precipitated by centrifuging the sample for 15 min at 20,000 × *g*. The DNA pellet was washed using ice cold 70% ethanol and centrifuged for 10 min at 20,000 × *g*. The pellet was air-dried and resuspended in 30 μl of MilliQ water. DNA was quantified with the Qubit dsDNA HS Assay Kit (Thermo Fisher Scientific, Waltham, MA) and sequenced on the Illumina sequencing platform. For library preparation the Nextera XT kit (Illumina, San Diego, California) was used according to the manufacturer’s instructions. Enzymatic tagmentation was performed starting with 1 ng of DNA, followed by incorporation of the indexed adapters and amplification of the library. After purification of the amplified library using AMPure XP beads (Beckman Coulter, Indianapolis), the two libraries were checked for quality and size distribution using the Agilent 2100 Bioanalyzer and the High sensitivity DNA kit. Quantitation of the libraries was performed by Qubit using the Qubit dsDNA HS Assay Kit (Thermo Fisher Scientific, Waltham, Massachusetts). The two libraries were pooled, denatured and sequenced with the Illumina Miseq sequence machine (San Diego, California). Paired end sequencing of 2 × 300 base pairs was performed using the MiSeq Reagent Kit v3 (Illumina, San Diego, California) according the manufacturers protocol. Sequencing resulted in a total of 55,685,154 and 56,928,602 reads for CTAB and PowerSoil extracted DNA, respectively.

### Genome Assembly, Binning, and Annotation

Reads were trimmed using BBDuk (BBMap), assembled by MEGAHIT v1.0.3 (Li et al., 2015) and binned using a combination of different algorithms, namely BinSanity (Graham et al., 2017), COCACOLA (Lu et al., 2017), CONCOCT (Alneberg et al., 2014), MaxBin 2.0 (Wu et al., 2016), and MetaBAT 2 (Kang et al., 2019). DAS Tool 1.0 was used for consensus binning (Sieber et al., 2018), and CheckM was used to assess the MAG quality (Parks et al., 2015). The average nucleotide identity using BLAST (ANIb) was calculated using JSpeciesWS software with standard settings (Richter et al., 2016). The genome of Bin 9 was integrated in the Microscope platform (Vallenet et al., 2006, 2009) and annotated as described elsewhere (Lücker et al., 2010).

### Phylogenetic Analysis

16S rRNA gene sequences were identified in the NCBI database by BLAST, aligned with the sequences obtained in this study by MUSCLE32 and used to build phylogenetic trees (with the Maximum Likelihood method and 500 bootstraps in Mega 7 (Kumar et al., 2016). Average Amino acid Identity (AAI) values were calculated using the AAI calculation tool developed by the Kostas lab (Rodriguez-R and Konstantinidis, 2014).

## Results and Discussion

### Proteobacterial and Verrucomicrobial Methanotrophs in the Soil of Favara Grande

The Favara Grande is an area on Pantelleria Island characterized by hydrothermal activity with gas emissions of CO_2_, H_2_, and CH_4_ (Picone et al., 2020). Within the bacterial community, methanotrophs belonging to the Gammaproteobacteria and Verrucomicrobia phyla could be identified through *pmoA* sequencing (Gagliano et al., 2014). 16S rRNA gene amplicon sequencing analysis, instead, did not detect Verrucomicrobia in the soil of Favara Grande, but potential methanotrophy was mainly attributed to Gammaproteobacteria (Gagliano et al., 2016). Recent Illumina metagenomic sequencing at a much higher resolution could show the presence of five MAGs related to methanotrophs (Picone et al., 2020; Supplementary Figure 1). MAG2 resembled a novel gammaproteobacterial *Methylobacter* species (Hogendoorn et al., 2021) and MAG8 and MAG16 were related to *Methylococcus* sp. The two remaining MAGs clustered within the phylum Verrucomicrobia. A detailed description of *Methylacidimicrobium thermophilum* AP8, an isolated representative of MAG5, was recently published (Picone et al., 2021). 16S rRNA analysis of MAG9 revealed a species phylogenetically distant to other known verrucomicrobial methanotrophs. The closest cultured relatives were *Methylacidiphilum* sp. RTK17 and *Methylacidiphilum infernorum* V4, that shared only 89.9% 16S rRNA identity to MAG9. This MAG was analyzed in detail.

**FIGURE 1:**
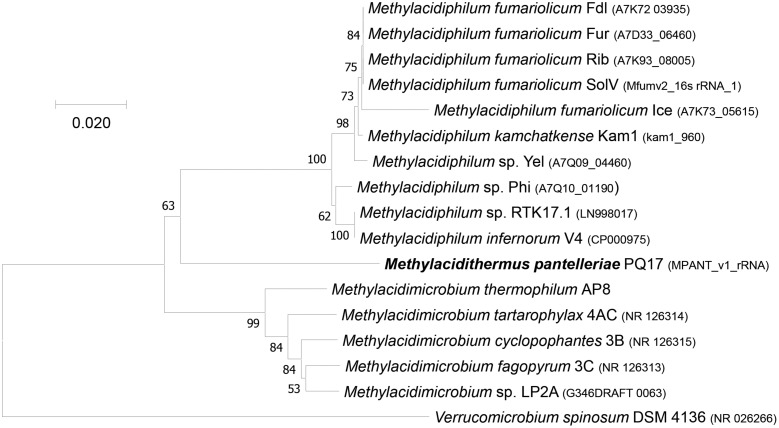
16S rRNA gene-based phylogenetic tree of methanotrophic Verrucomicrobia. The evolutionary history was inferred using the Neighbor-Joining method. The optimal tree with the sum of branch length = 0.41438253 is shown. The percentage of replicate trees (>50%) in which the associated taxa clustered together in the bootstrap test (500 replicates) are shown next to the branches. The tree is drawn to scale, with branch lengths in the same units as those of the evolutionary distances used to infer the phylogenetic tree. The evolutionary distances were computed using the Maximum Composite Likelihood method and are in the units of the number of base substitutions per site. The analysis involved 17 nucleotide sequences. All ambiguous positions were removed for each sequence pair. There were a total of 1,575 positions in the final dataset. Evolutionary analyses were conducted in MEGA7 (Kumar et al., 2016).

Phylogenetic analysis showed that the 16S rRNA gene of MAG9 clustered in between *Methylacidimicrobium* and *Methylacidiphilum* species (Figure 1; Dunfield et al., 2007; Pol et al., 2007; Op den Camp et al., 2009; van Teeseling et al., 2014; Schmitz et al., 2021). The 16S rRNA and AAI values (Table 1 and Supplementary Table 1) fell below the threshold for species delimitation (95% for AAI and 98.7–99% for 16S rRNA) (Stackebrandt and Ebers, 2006; Thompson et al., 2013). Considering the AAI thresholds proposed by Konstantinidis et al. (2017) for uncultivated microorganisms (45–65% for the same family, 65–95% for the same genus and 95–100% for the same species), these results classified MAG9 as representing a new species of a new genus, for which we propose the name “*Candidatus* Methylacidithermus pantelleriae” sp. PQ17. This “*Ca.* Methylacidithermus” genus is the third genus of methanotrophic Verrucomicrobia within the family Methylacidiphilaceae, that adds to the previously described *Methylacidiphilum* and *Methylacidimicrobium* genera (Op den Camp et al., 2009; van Teeseling et al., 2014; Schmitz et al., 2021).

**TABLE 1:** Average amino acid identity (AAI) value comparison between different verrucomicrobial methanotroph species.

**Species**	**1**	**2**	**3**	**4**	**5**	**6**	**7**
1. “*Ca*. Methylacidithermus pantelleriae” PQ17		**50.6**	**50.8**	**51.0**	**53.6**	**53.3**	**52.7**
2. *Methylacidiphilum fumariolicum* SolV	**50.6**		75.3	94.2	54.6	54.8	54.6
3. *Methylacidiphilum infernorum* V4	**50.8**	75.3		75.4	55.1	55.1	54.6
4. *Methylacidiphilum kamchatkensis Kam1*	**51.0**	94.2	75.4		55.1	55.2	54.8
5. *Methylacidimicrobium thermophilum* AP8	**53.6**	54.6	55.1	55.1		78.0	78.5
6. *Methylacidimicrobium cyclopopanthes* 3C	**53.3**	54.8	55.1	55.2	78.0		79.9
7. *Methylacidimicrobium tartarophylax* 4AC	**52.7**	54.6	54.6	54.8	78.5	79.9	

*Values are expressed in %. In bold, the comparison of strain PQ17 with other verrucomicrobial methanotrophs.*

### Genomic Characterization of “*Ca.* Methylacidithermus Pantelleriae”

The draft genome of strain PQ17 was analyzed in details to gain a better understanding about its metabolic potential and its role in the geothermal soil of Pantelleria. MAG9 consisted of 71 contigs ranging from 401,379 to 2,075 bp, for a total of 2,466,655 bp, containing 3,127 predicted CDSs and an overall 55.2% GC-content. CheckM analysis revealed a completeness of 98.6, 1.3% contamination and no strain heterogeneity (Supplementary Figure 2). A total of 3,204 genes could be identified, 3,127 of which were protein coding genes and 77 were RNA genes. One 16S and two 23S and 5S rRNA genes were retrieved, indicating that one 16S rRNA copy was probably missing from the draft genome. Functions could be assigned to 2,164 protein coding genes (Table 2). 47.4% of the predicted genes were allocated into Clusters of Orthologous Groups (Supplementary Table 2).

**TABLE 2:** Genome statistics of “*Ca*. Methylacidithermus pantelleriae” PQ17.

**Attribute**	**Value**
Genome size (bp)	2,466,655
DNA coding (bp)	2,037,457
DNA G + C (%)	55.2%
DNA scaffolds	71
Total genes	3,204
Protein coding genes	3,127
RNA genes	77
rRNA genes	5
tRNA genes	65
Pseudo genes	8
Genes in internal clusters	–
Genes with function prediction	1,231
Genes assigned to COGs	1,482

**FIGURE 2:**
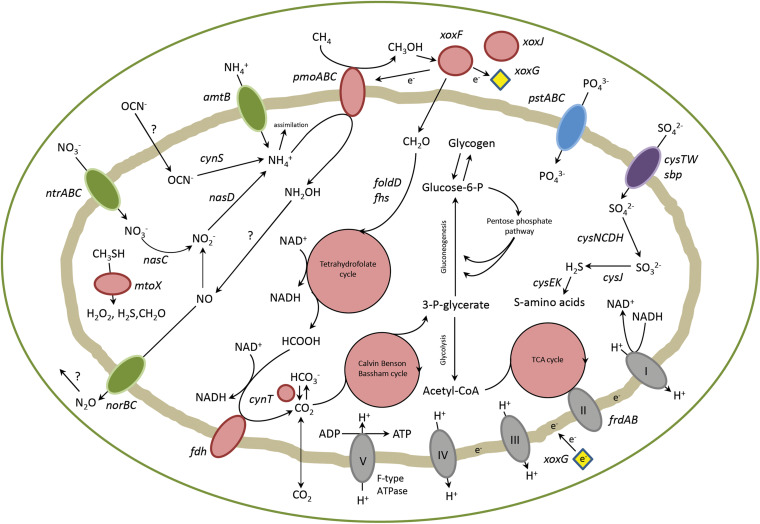
Overview of metabolic pathways in “*Ca.* M. pantelleriae.” Colors of enzymes and transporters indicate nitrogen metabolism (green), carbon metabolism (red), sulfate metabolism (purple), phosphate metabolism (blue) and complexes of the respiratory chain (gray). Genes: *pmoABC*, methane monoxygenase; *xoxF*, methanol dehydrogenase, *xoxG*, cytochrome c_*L*_; *xoxJ*, periplasmic binding protein; *folD*, bifunctional 5,10-methylene-tetrahydrofolate dehydrogenase/5,10-methylene-tetrahydrofolate cyclohydrolase; *fhs*, formate-tetrahydrofolate ligase; *fdh*, formate dehydrogenase; *cynT*, carbonic anhydrase; *frdAB*, fumarate reductase; *pstABC*, phosphate transporter; *cysTW/sbp*, sulfate transporter; *amtB*, ammonia transporter; *ntrABC*, nitrate transporter; *nasC*, assimilatory nitrate reductase; *nasD*, assimilatory nitrite reductase; *norBC*, nitric oxide reductase; *cynS*, cyanase; *cysCDH*, adenylyl-sulfate kinase, sulfate adenylyltransferase, phosphoadenosine phosphosulfate reductase; *cysJ*, sulfite reductase; *cysK*, cysteine synthase; *cysE*, serine acetyltransferase; *mtoX*, methanethiol oxidase.

Genes involved in carbon, nitrogen and sulfur metabolism were analyzed in detail. Their pathways will be described in the upcoming sections and a schematic representation of the metabolism of strain PQ17 can be found in Figure 2.

### Methanotrophy

As a first step in methane oxidation, methane monooxygenases use molecular oxygen to break the energetically strong C-H bond of methane to form methanol (Sirajuddin and Rosenzweig, 2015). So far, two types of this enzyme have been described: a membrane-bound pMMO and a soluble methane monooxygenase (sMMO). One single *pmoCAB* operon and a *pmoD* subunit, encoding a copper-binding protein (Fisher et al., 2018), were found in MAG9, whereas no other *mmo* genes were identified (Table 3). This is in line with previously described verrucomicrobial methanotrophs, although the number of *pmo* operons seems to be variable (Op den Camp et al., 2009; van Teeseling et al., 2014; Erikstad et al., 2019; Picone et al., 2020; Schmitz et al., 2021). The PmoA phylogenetic tree including strain PQ17 (Supplementary Figure 3) supports the phylogeny derived from 16S rRNA gene analysis.

**TABLE 3:** Genes encoding for enzymes involved in methane oxidation pathway, along with their Enzyme Commission (EC) numbers and percentage identity to the most similar homologue.

**Enzyme**	**Identifier**	**EC number**	**Gene**	**Identity (%)**	**Species**
Particulate methane monooxygenase	*MPNT_60061*	1.14.18.3	*pmoA*	62.7	*M. infernorum* V4
	*MPNT_60059*		*pmoB*	47.3	*M. infernorum* V4
	*MPNT_60062*		*pmoC*	59.2	*M. infernorum* V4
	*MPNT_60058*		*pmoD*	35.9	*M. infernorum* V4
Methanol dehydrogenase	*MPNT_10387*	1.1.99.8	*xoxF*	74.7	*M. fumariolicum SolV*
Cytochrome C_*L*_	*MPNT_10390*		*xoxG*	41.7	*M. fumariolicum SolV*
Putative periplasmic binding protein	*MPNT_10389*		*xoxJ*	55.4	*M. infernorum* V4
Putative TonB-dependent receptor	*MPNT_80102*		*cirA*	51.4	*M. ishizawai*
ABC transporter ATP-binding protein	*MPNT_20035*			54.5	*Verrucomicrobia Tous-C9LFEB*
Methenyltetrahydrofolate cyclohydrolase/methylenetetrahydrofolate dehydrogenase	*MPNT_320016*	3.5.4.9/1.5.1.5	*folD*	57.7	*M. infernorum* V4
Formate-tetrahydrofolate ligase	*MPNT_330006*	6.3.4.3	*fhs*	56.9	*M. marinum*
Formate dehydrogenase	*MPNT_130014*	1.17.1.9	*fdh*	77.2	*C. sequanensis*
Formate dehydrogenase	*MPNT_310008*	1.2.1.2	*fdsA*	77.3	*N. kurashikiensis*
	*MPNT_310010*		*fdsB*	69.1	*M. ishizawai*
	*MPNT_310011*		*fdsC*	62.1	*Rhizobium*
	*MPNT_310006*		*fdsD*	54.8	*Burkholderiales* bacterium

The second step in methane oxidation is the conversion of methanol to formaldehyde or formate. Two types of pyrroloquinoline quinone (PQQ)-containing MDHs are generally found in methanotrophs and methylotrophs: The MxaFI and the XoxF type MDH. Whereas MxaFI depends on calcium for its catalysis, XoxF was found to contain lanthanides in the active site (Pol et al., 2014). Analysis of the MDH sequence of strain PQ17 showed that this enzyme presented a conserved Asp residue required for the coordination of lanthanides in the active site (Keltjens et al., 2014; Pol et al., 2014; Good et al., 2020). Furthermore, it exhibited 74% amino acid identity to XoxF from *Methylacidiphilum fumariolicum* SolV, confirming that this protein was a XoxF type and it belonged to group XoxF1 (Keltjens et al., 2014). *xoxG* and *xoxJ* genes were also found in the genome of strain PQ17 (Table 3). *xoxG* encodes a cytochrome C_*L*_ that functions as electron acceptor for XoxF, whereas *xoxJ* encodes a periplasmic binding protein that is proposed to be involved in the activation of XoxF and, more specifically, in the insertion of the PQQ cofactor in apo-XoxF (Zheng et al., 2018; Featherston et al., 2019; Versantvoort et al., 2019). In the *Methylacidiphilum* species SolV and Kam1 these proteins are exceptionally present as the fusion protein XoxG/J (Islam et al., 2008; Versantvoort et al., 2019).

Several genes have been proposed as candidates for lanthanide incorporation in bacterial cells (Ochsner et al., 2019). The gene *cirA*, encoding a TonB-dependent receptor, and a component of the ABC transport system have been identified in other Verrucomicrobia (Picone et al., 2021) and were also shown to be present in the genome of strain PQ17 (Table 3). The lanthanide binding protein lanmodulin described in *M. extorquens*, instead, could not be found (Cotruvo et al., 2018).

XoxF from strain SolV is known to convert methanol to formate *in vitro* in a four electron process (Pol et al., 2014). In *Methylobacterium extorquens* AM instead, XoxF generated formaldehyde (Good et al., 2018), which is converted to formate by formaldehyde dehydrogenase. Similarly to strain SolV, no formaldehyde dehydrogenase was detected in “*Ca.* M. pantelleriae.” If formaldehyde is produced, different detoxification pathways are known. The tetrahydrofolate pathway was the only pathway identified in strain PQ17. The first step in this cycle is a spontaneous reaction which couples formaldehyde to tetrahydrofolate forming 5,10-methylenetetrahydrofolate. The bifunctional enzyme FolD catalyzes the second and third steps of this cycle, converting 5,10-methylenetetrahydrofolate via 5,10-methenyltetrahydrofolate to 10-formyltetrahydrofolate. The last reaction step is catalyzed by formate tetrahydrofolate ligase (Fhs), in which formate is produced and tetrahydrofolate is regenerated (Table 3 and Figure 2; Vorholt, 2002). Formaldehyde could also be produced by methanethiol oxidase (MtoX, MPNT_180031), an enzyme apparently conserved in verrucomicrobial methanotrophs (Eyice et al., 2018; Picone et al., 2021).

In the last step of methane oxidation, formate is converted to CO_2_ by formate dehydrogenase (FDH). In the bacterial kingdom, a large variety of FDH exist, which are all highly diverse regarding cofactor usage and mechanism (Hartmann et al., 2015). For “*Ca.* M. pantelleriae,” two different FDHs were found in the genome, one was cytoplasmic and the other was predicted to be a membrane-bound enzyme complex composed of four subunits (Table 3).

### Central Carbon Metabolism

Methanotrophs assimilate carbon into their metabolism using different pathways. Verrucomicrobia are generally able to fix CO_2_ via the Calvin-Benson-Bassham (CBB) cycle (Khadem et al., 2011). This cycle is initiated by the reaction of carbon dioxide with ribulose bisphosphate, which is catalyzed by ribulose bisphosphate carboxylase (RuBisCO) (Tabita, 2007). The small and a large subunit of this enzyme could be identified in the genome, together with two carbonic anhydrases (Table 4). The products of RuBisCO are two molecules of 3-phosphoglycerate (3-PG), which are converted back to ribulose bisphosphate through a series of gluconeogenic and pentose phosphate pathway reactions. Every three CO_2_ molecules yield net one molecule of 3-PG, which can be incorporated in central carbon metabolism.

**TABLE 4:** Key enzymes for three major carbon assimilation pathways in methanotrophs.

**Enzyme**	**Identifier**	**EC number**	**Gene**	**Identity (%)**	**Species**
Ribulose-1,5-bisphosphate carboxylase/oxygenase (RuBisCO)^a^	*MPNT_100035*	4.1.1.39	*cbbS*	64.4	*Gemmatimonadetes* bacterium
	*MPNT_100036*		*cbbL*	84.4	*M. infernorum V4*
RuBisCo-like protein	*MPNT_20090*			47.7	*T. mobilis*
Carbonic anhydrase 1	*MPNT_100082*	4.2.1.1	*mtcA1*	76.8	*NC10 bacterium*
Carbonic anhydrase 2	*MPNT_210022*	4.2.1.1	*mtcA2*	79.3	*Verrucomicrobia* bacterium
Serine hydroxymethyltransferase^b^	*MPNT_220042*	2.1.2.1	*glyA*	60.4	*M. infernorum* V4
Serine-glyoxylate aminotransferase^b^	*MPNT_60063*	2.6.1.45	*sgaA*	68.5	*M. kamchatkense* Kam1

*^a^CBB cycle. ^b^Serine cycle. And their respective Enzyme Commission (EC) numbers, and percentage identity to the most similar homologue.*

Beside the CBB cycle, the Serine and the RuMP pathways are other strategies for carbon incorporation in microorganisms (Chistoserdova, 2011). Some genes involved in the Serine pathway could be found in the genome of strain PQ17 (Table 4), but four essential genes were lacking (*hprA* EC 1.1.1.29, *gckA* 2.7.1.165, *mtkB* EC 6.1.2.9, *mcl* EC 4.1.3.24). Likewise, two genes required for the RuMP pathway were also absent (*hxlA* EC 4.1.2.43, *hxlB* EC 5.3.1.27). Therefore, it is highly unlikely for “*Ca.* M. pantelleriae” to fix carbon using these pathways.

All glycolytic, gluconeogenic and pentose phosphate pathway genes could be retrieved from MAG9 (Supplementary Table 3), except for phosphofructokinase (EC 2.7.1.11). The other two genera of verrucomicrobial methanotrophs, *Methylacidiphilum* and *Methylacidimicrobium* have genes encoding this protein. All genes for the citric acid cycle were found. Moreover, genes for synthesis and degradation of glycogen were identified.

### Energy Conservation and Respiration

“*Ca*. M. pantelleriae” uses O_2_ as electron acceptor. Complex I of the respiratory chain (*nuoABCDEFGHIKLMN*) was found in the genome. For complex II, subunits a, b and c of succinate dehydrogenase were found, but subunit d was lacking. Subunits for a canonical complex III could not be retrieved. The verrucomicrobial methanotrophs, including strain PQ17, possess genes encoding an Alternative Complex III, a structurally different protein complex with similar function (MPNT_10279-10285) (Refojo et al., 2012; Schmitz et al., 2021). Finally, the electrons are transferred to complex IV and the F_0_F_1_ ATP synthase (complex V) generating ATP using the proton motive force (Supplementary Table 3). “*Ca*. M. pantelleriae” possesses genes encoding two distinct Complexes IV: aa3-type and a ba3-type. The genomes of the other verrucomicrobial methanotrophs encode for an additional cbb3-type Complex IV (Schmitz et al., 2021).

### Amino Acid Biosynthesis

Pathways for the biosynthesis of 12 amino acids (alanine, isoleucine, leucine, proline, valine, phenylalanine, tyrosine, tryptophan, arginine, lysine, threonine, and cysteine) were completely present in the genome. For histidine, only one gene encoding the biosynthesis protein HisE was absent.

The complete pathways for asparagine/aspartate and glutamine/glutamate biosynthesis could not be fully resolved but the enzyme for the conversion of oxaloacetate to aspartate was identified (*aspC* MPNT_250010). Genes encoding enzymes for the formation of asparagine, instead, could not be retrieved. Glutamate could be formed from 2-oxoglutarate via glutamate synthase (GltB, MPNT_40080) or from gamma-aminobutyric acid through glutamate decarboxylase (MPNT_510001). Glutamate dehydrogenase (GDH) was not identified, whereas glutamine synthetase and glutamate synthase (GS-GOGAT) were both present (Supplementary Table 3). GDH and GS-GOGAT pathways are also used for ammonia incorporation into biomass. Ammonia incorporation via GS-GOGAT usually happens under low ammonia concentrations (Tyler, 1978; Bellion and Bolbot, 1983). The presence of an alanine dehydrogenase (MPNT_50137) in the genome indicates that ammonia could also be incorporated though alanine, starting from pyruvate and NH_4_^+^.

The pathways for glycine and serine biosynthesis are less straightforward. If serine is synthetized from 3-PG, only D-3-phosphoglycerate dehydrogenase/2-oxoglutarate reductase was present (*serA*, MPNT_20138), whereas phosphoserine aminotransferase (*serC*, EC 2.6.1.52) and phosphoserine phosphatase (*serB*, EC 3.1.3.3) were absent. We cannot exclude that these reactions are still performed *in vivo*, but catalyzed by unknown enzymes. The fragmentation of the genome could also prevent us from retrieving these genes. Assuming that these pathways are actually missing in strain PQ17, serine can still be synthesized in a one-step reaction catalyzed by serine hydroxymethyltransferase (*glyA*, MPNT_220042) using 5,10-methylenetetrahydrofolate and glycine. For this to be a feasible strategy, “*Ca*. M. pantelleriae” should be able to synthesize its glycine from a different source than serine. As all four subunits of the glycine cleavage system are present in its genome (MPNT_20097, 20098, 20099, 420008), we propose that “*Ca*. M. pantelleriae” could use this machinery in reverse to synthesize glycine from ammonia, carbon dioxide and 5,10-methylenetetrahydrofolate (Kikuchi et al., 2008), which has also been described previously for *Clostridium acidiurici* (Gariboldi and Drake, 1984). Furthermore, glycine can be synthetized from glyoxylate (*agxt2*, MPNT_100077) and from sarcosine (*dauA*, MPNT_10078).

### Nitrogen Metabolism

Ammonium from the environment can be imported directly into the cell using either of two AmtB transporters (MPNT_100073, 250005). Alternatively, nitrogen can be obtained by uptake of nitrate via a NrtABC transporter (Supplementary Table 3), followed by reduction to ammonia by NasC and NasD (Table 5 and Supplementary Table 3). Interesting is the presence of the gene *cynS*, which encodes cyanase, an enzyme converting cyanate and bicarbonate to carbon dioxide and ammonium. Cyanate can act as energy and nitrogen source for nitrifiers (Palatinszky et al., 2015) and its presence has been detected in other verrucomicrobial methanotrophs (Picone et al., 2021). Unlike other verrucomicrobial methanotrophs, genes encoding a nitrogenase enzyme could not be found (Op den Camp et al., 2009; Khadem et al., 2010; Schmitz et al., 2021). Fixed ammonium is mostly used for biosynthetic purposes, but some ammonium is also converted into hydroxylamine by *pmoA*, which is a structural homolog of ammonium monooxygenase *amoA* (Sirajuddin and Rosenzweig, 2015). As hydroxylamine is toxic to the cell, it must be further metabolized into less harmful compounds. However, hydroxylamine oxidoreductase (*hao*), which is present in ammonia oxidizers but also in other methanotrophs such as *M. fumariolicum* SolV (Pol et al., 2007), could not be retrieved from the MAG. The nitric oxide reductase encoded by *norBC* (Table 5) was identified, whereas other denitrification genes, like *narB* (EC 1.7.5.1) and *nosZ* (EC 1.7.2.5), were not detected.

**TABLE 5:** Genes encoding for enzymes involved in nitrogen metabolism, along with their Enzyme Commission (EC) numbers, and percentage identity to the most similar homologue.

**Enzyme**	**Identifier**	**EC number**	**Gene**	**Identity (%)**	**Species**
Assimilatory nitrate reductase	*MPNT_110017*	1.7.1.1	*nasC*	57.1	*P. methylaliphatogenes*
Nitrite reductase	*MPNT_110016*	1.7.1.4	*nasD*	46.3	*P. methylaliphatogenes*
Cyanate hydratase	*MPNT_50182*	4.2.1.104	*cynS*	64.1	*K. tusciae*
Nitric oxide reductase	*MPNT_410005*	1.7.2.5	*norB*	72.9	*O. profundus*
	*MPNT_410004*		*norC*	73.4	*O. profundus*

### Sulfur and Phosphate Metabolism

The primary way to fix sulfur for “*Ca*. M. pantelleriae” is to reduce sulfate to biologically available sulfide. For this, sulfate needs to be transported into the cell using the sulfate ABC-transporter sbp/cysTW (MPNT_580001-580004). Subsequently, sulfate can be reduced to sulfite via adenylyl sulfate and 3′-phosphoadenylyl sulfate intermediates by the genes, catalyzed by *cysD* (MPNT_10354), *cysC* (MPNT_10355) and *cysH* (MPNT_20190), respectively. Finally, sulfite can be further reduced to H_2_S by *cysJ* (MPNT_10061, MPNT_40049) or *sir1* (MPNT_20189) and used for cysteine biosynthesis (*cysK* (MPNT_110064). Phosphate can be transported directly over the membrane using the ABC-transporter encoded by *pstABCS* (Supplementary Table 3) and does not require further conversions. The presence of polyphosphate particles has been observed in verrucomicrobial methanotrophs (van Teeseling et al., 2014). Polyphosphate storage is likely in strain PQ17 as genes encoding polyphosphate kinase (MPNT_190035) and exopolyphosphatase (MPNT_40183) were identified.

## Conclusion and Ecological Role

“*Ca.* Methylacidithermus pantelleriae” PQ17 presents most of the typical characteristics of verrucomicrobial methanotrophs. This microorganism was detected in a thermoacidophilic volcanic environment and its genome predicts it to be an aerobic bacterium able to fix carbon via the CBB cycle. Methane oxidation to methanol may use the methane monooxygenase encoded by the *pmoCAB* operon and the conversion of methanol could be carried out by the XoxF-type MDH. Contrary to other verrucomicrobial methanotrophs, the genome of strain PQ17 does not encode genes for nitrogen fixation, nor for the oxidation of hydrogen, a common energy substrate for verrucomicrobial methanotrophs. These features, together with phylogenetic analysis, suggest that “*Ca.* M. pantelleriae” has evolved differently from other verrucomicrobial methanotrophs. This bacterium probably utilizes exclusively methane or methanol for energy production and provides nitrogen for biomass mainly via nitrate and ammonia and not by fixing N_2_ gas.

## Data Availability Statement

The datasets presented in this study can be found in online repositories. The names of the repository/repositories and accession number(s) can be found below: https://www.ncbi.nlm.nih.gov/genbank/, PRJEB38823 (genome accession number CAJNOB000000000), https://mage.genoscope.cns.fr/microscope/mage/viewer.php?O_id=8619, 8619.

## Author Contributions

NP, AP, MJ, and HO designed the projects and experiments. NP, CH, AP, AG, WD’A, PQ, and HO sampled the geothermal soils. NP and CH performed the DNA isolation. TA, JF, NP, and PB performed sequencing, assembly and annotation. NP, PB, AP, and HO carried out the data analysis. NP, PB, and HO wrote the manuscript. All authors contributed to revision of the manuscript, and read and approved the submitted version.

## Conflict of Interest

The authors declare that the research was conducted in the absence of any commercial or financial relationships that could be construed as a potential conflict of interest.
