# Supplementary material for: Metagenome Assembled Genome of a Novel Verrucomicrobial Methanotroph From Pantelleria Island
Source: Front Microbiol. 2021 May 19;12:666929. doi: 10.3389/fmicb.2021.666929 (PMC8170126; doi:10.3389/fmicb.2021.666929)
Supplement: Supplementary file 1 [file Data_Sheet_1.PDF]

Supplementary Material

Metagenome assembled genome of a novel verrucomicrobial methanotroph from Pantelleria Island

Nunzia Picone[1], Pieter Blom[1], Carmen Hogendoorn[1], Jeroen Frank[1], Theo van Alen[1], Arjan Pol[1], Antonia L. Gagliano[2], Mike S.M. Jetten[1], Walter D'Alessandro[2], Paola Quatrini[3] and Huub J.M. Op den Camp[1]*

[1]Department of Microbiology, Institute for Water and Wetland Research, Radboud University, Nijmegen, the Netherlands

[2]Istituto Nazionale di Geofisica e Vulcanologia (INGV), Palermo, Italy

[3]Department of Biological, Chemical and Pharmaceutical Sciences and Technologies (STEBICEF), University of Palermo, Palermo, Italy

*** Correspondence:** h.opdencamp@science.ru.nl

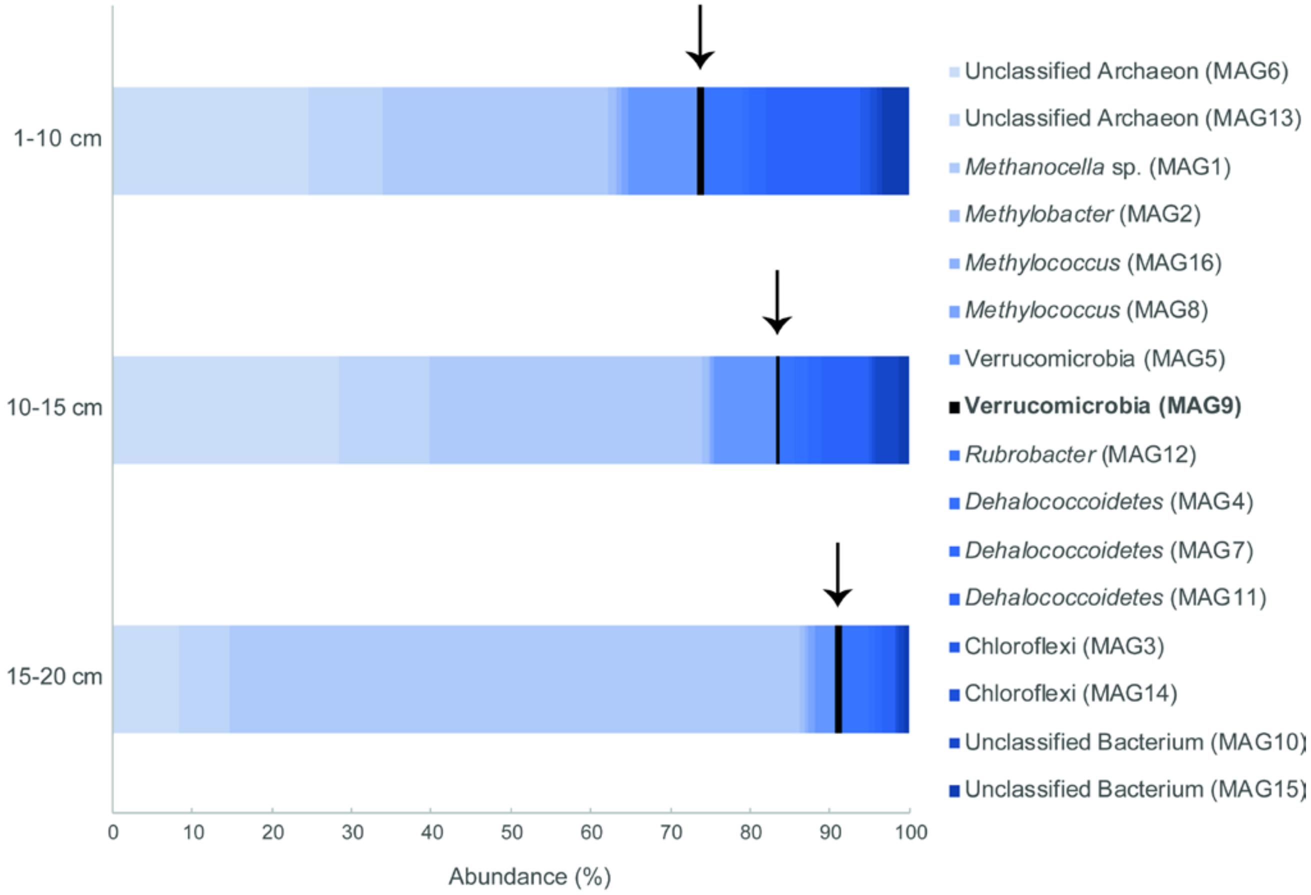

Supplementary Figure S1 | Relative abundance of the different MAGs binned from the Favare Grande metagenome (completeness >95 %) at three different depths in the soil. The relative abundance of Verrucomicrobia (MAG9, in black) is 0.8% in the top layer, 0.4% at 10-15 cm and 0.9% at 15-20 cm. The black arrow refers to MAG9.

Supplementary Table S1 | Calculated % identity between the 16S rRNA genes of verrucomicrobial methanotrophs**.**

	Species	1	2	3	4	5	6	7	8	9	10	11	12	13	14	15	16
1	*Methylacidiphilum_fumariolicum*_Fdl_(A7K72_03935)																
2	*Methylacidiphilum_fumariolicum*_Fur_(A7D33_06460)	100.0															
3	*Methylacidiphilum_fumariolicum*_Ice_(A7K73_05615)	98.1	98.1														
4	*Methylacidiphilum_fumariolicum*_Rib_(A7K93_08005)	100.0	100.0	98.1													
5	*Methylacidiphilum_fumariolicum*_SolV_(Mfumv2_16s_rRNA_1)	100.0	100.0	98.1	100.0												
6	*Methylacidiphilum_kamchatkense*_Kam1_(kam1_960)	99.7	99.7	97.8	99.7	99.7											
7	*Methylacidiphilum_infernorum*_V4_(CP000975)	98.5	98.5	96.5	98.5	98.5	98.5										
8	*Methylacidiphilum*_sp._Phi_(A7Q10_01190)	98.5	98.5	96.6	98.5	98.5	98.6	99.0									
9	*Methylacidiphilum*_sp._RTK17.1_(LN998017)	98.5	98.5	96.5	98.5	98.5	98.5	100.0	99.0								
10	*Methylacidiphilum*_sp._Yel_(A7Q09_04460)	99.4	99.4	97.5	99.4	99.4	99.4	98.4	98.5	98.4							
11	*Methylacidimicrobium_thermophilum*_AP8_(MTHMO_RRNA1)	89.8	89.8	88.3	89.8	89.8	89.9	90.0	90.0	90.0	89.6						
12	*Methylacidimicrobium_tartarophylax*_4AC_(NR_126314)	89.8	89.8	88.2	89.8	89.8	89.7	90.0	90.4	90.0	89.3	95.6					
13	*Methylacidimicrobium_fagopyrum*_3C_(NR_126313)	89.5	89.5	88.0	89.5	89.5	89.4	89.8	90.0	89.8	89.0	96.4	97.3				
14	*Methylacidimicrobium*_sp._LP2A_(G346DRAFT_0063)	89.5	89.5	87.8	89.5	89.5	89.5	89.9	89.9	89.9	89.0	96.9	97.3	98.1			
15	*Methylacidimicrobium_cyclopophantes*_3B_(NR_126315)	89.7	89.7	88.2	89.7	89.7	89.6	89.8	89.7	89.9	89.2	97.0	97.1	97.7	98.1		
16	*Methylacidithermus_pantelerii*_PQ17_MPANT_v1_rRNA	89.1	89.1	87.6	89.1	89.1	89.2	89.6	89.7	89.6	89.3	89.3	88.7	89.0	88.7	88.5	

Analyses were conducted using the Jukes-Cantor model. The analysis involved 16 nucleotide sequences. All ambiguous positions were removed for each sequence pair. There were a total of 1575 positions in the final dataset.

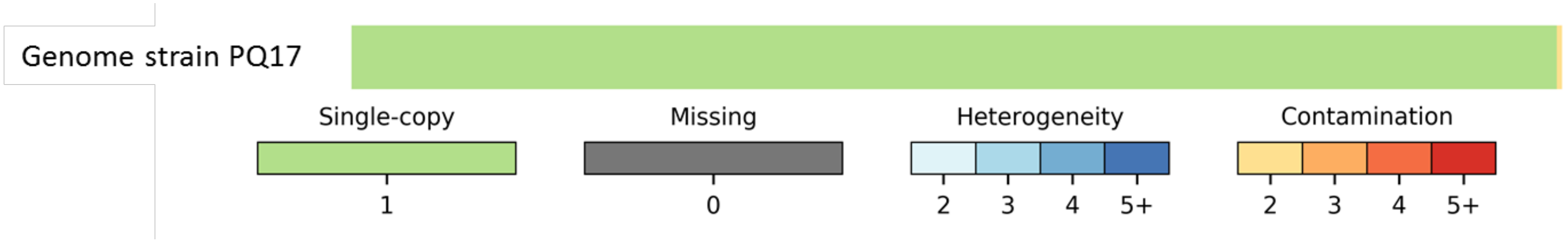

Supplementary Figure S2 | CheckM analysis of the draft genome from "Ca. Methylacidithermus pantelleria PQ17. Completeness 98.6%, contamination 1.3%, heterogeneity 0%.

Supplementary Table S2 | Number of genes associated with general COG functional category prediction.

Class ID	Description	CDS	%
D	Cell cycle control, cell division, chromosome partitioning	25	0.8
M	Cell wall/membrane/envelope biogenesis	149	4.8
N	Cell motility	8	0.3
O	Post-translational modification, protein turnover, chaperones	76	2.4
T	Signal transduction mechanisms	37	1.2
U	Intracellular trafficking, secretion, and vesicular transport	41	1.3
V	Defense mechanisms	32	1.0
B	Chromatin structure and dynamics	1	0.03
J	Translation, ribosomal structure and biogenesis	132	4.2
K	Transcription	68	2.2
L	Replication, recombination and repair	124	4.0
C	Energy production and conversion	110	3.5
E	Amino acid transport and metabolism	162	5.2
F	Nucleotide transport and metabolism	48	1.5
G	Carbohydrate transport and metabolism	108	3.5
H	Coenzyme transport and metabolism	100	3.2
I	Lipid transport and metabolism	53	1.7
P	Inorganic ion transport and metabolism	105	3.4
Q	Secondary metabolites biosynthesis, transport and catabolism	45	1.4
R	General function prediction only	209	6.7
S	Function unknown	103	3.3
-	Not in COGs	1645	52.6

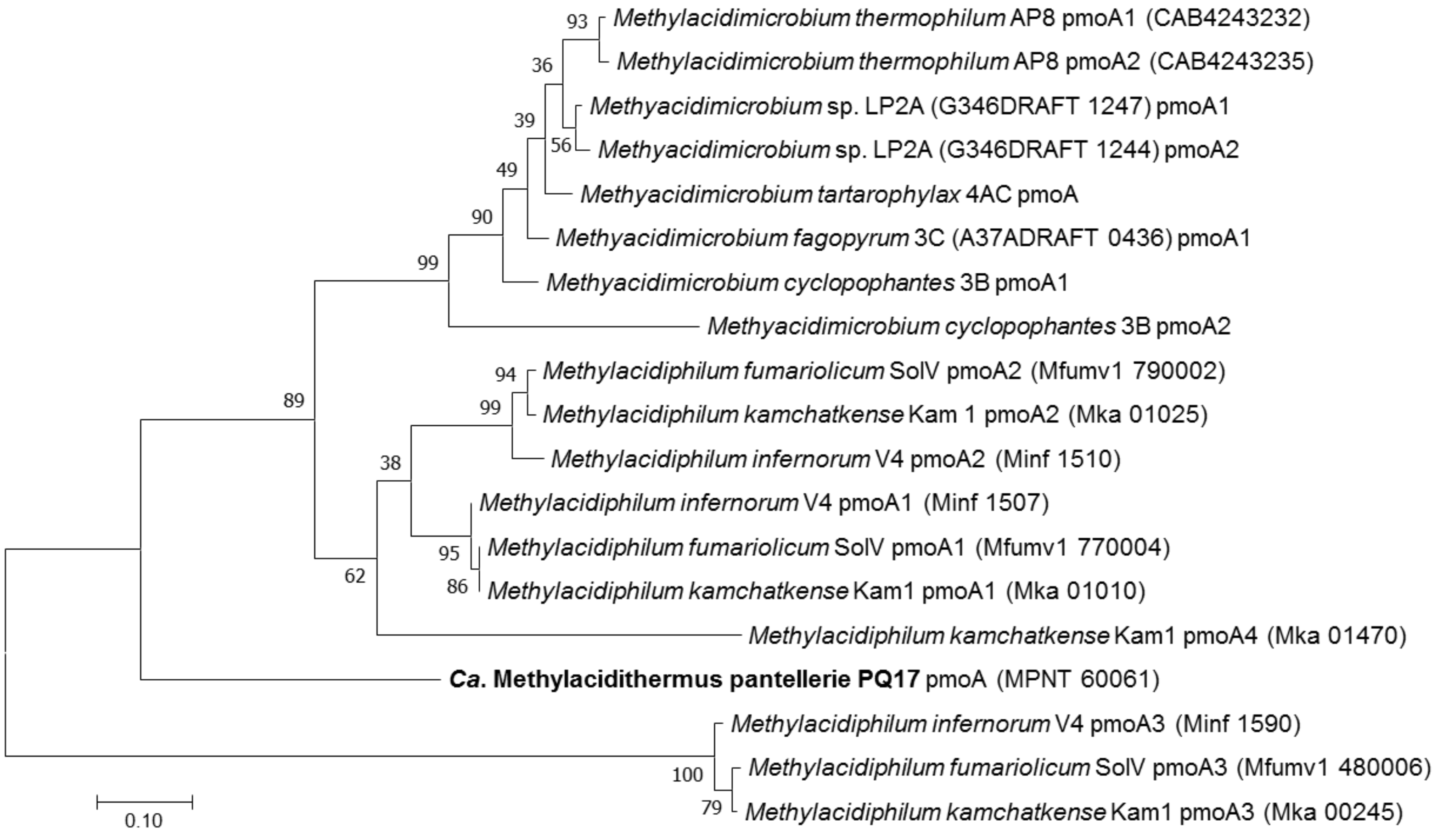

Supplementary Figure S3 | Molecular phylogenetic analysis by Maximum Likelihood method of verrucomicrobial PmoA protein sequences. The evolutionary history was inferred by using the Maximum Likelihood method based on the JTT matrix-based model. The tree with the highest log likelihood (-3227.12) is shown. The percentage of trees in which the associated taxa clustered together is shown next to the branches. Initial tree(s) for the heuristic search were obtained automatically by applying Neighbor-Join and BioNJ algorithms to a matrix of pairwise distances estimated using a JTT model, and then selecting the topology with superior log likelihood value. The tree is drawn to scale, with branch lengths measured in the number of substitutions per site. The analysis involved 19 amino acid sequences. All positions containing gaps and missing data were eliminated. There were a total of 243 positions in the final dataset.

Supplementary Table S3 | “*Candidatus* Methylacidithermus pantelleriae PQ17” genes with predicted function.

**Pentose phosphate pathway**			
**Identifier**	**Gene**	**Product**	**E.C. number**
MPNT_130008	*zwf*	Glucose-6-phosphate 1-dehydrogenase	1.1.1.49
MPNT_20210	*pgl*	6-Phosphogluconolactonase	3.1.1.31
MPNT_20211	*pgd*	6-Phosphogluconate dehydrogenase, decarboxylating	1.1.1.44
MPNT_30179	*gnd*	NAD-dependent 6-phosphogluconate dehydrogenase	1.1.1.343
MPNT_10335	*rpe1*	Ribulose-phosphate 3-epimerase	5.1.3.1
MPNT_70076	*rpe2*	D-ribulose-5-phosphate 3-epimerase	5.1.3.1
MPNT_100032	*tkt*	Transketolase	2.2.1.1
MPNT_100038	*tal*	Transaldolase	2.2.1.2
**Glycolysis/Gluconeogenesis**			
**Identifier**	**Gene**	**Product**	**E.C. number**
MPNT_30180	*pgi*	Glucose-6-phosphate isomerase	5.3.1.9
MPNT_110043	*fba*	Fructose-bisphosphate aldolase	4.1.2.13
MPNT_120045	*tpiA*	Triosephosphate isomerase	5.3.1.1
MPNT_120047	*gapA*	glyceraldehyde-3-phosphate dehydrogenase	1.2.1.12
MPNT_120046	*pgk*	Phosphoglycerate kinase	2.7.2.3
MPNT_20205	*gpmA*	Phosphoglycerate mutase (2,3-diphosphoglycerate dependent)	5.4.2.11
MPNT_100031	*phoE*	Phosphoglycerate mutase, PhoE family	5.4.2.12
MPNT_10145	*eno*	Enolase	4.2.1.11
MPNT_240025	*pykF*	Pyruvate kinase	2.7.1.40
MPNT_10240	*pycA*	Pyruvate carboxylase, biotin carboxylase	6.3.4.14
MPNT_20025	*ppc*	Phosphoenolpyruvate carboxylase	4.1.1.31

MPNT_180025	*glpX*	Fructose-1,6-bisphosphatase	3.1.3.11
**Citric acid cycle**			
**Identifier**	**Gene**	**Product**	**E.C. number**
MPNT_10240	*pycA*	Pyruvate carboxylase, biotin carboxylase	6.3.4.14
MPNT_420010	*pdhC*	Pyruvate dehydrogenase, subunit gamma (acetyltransferase component)	1.2.4.1
MPNT_420011	*pdhB*	Pyruvate dehydrogenase, subunit beta	1.2.4.1
MPNT_420012	*pdhA*	Pyruvate dehydrogenase, subunit alpha (E1 component)	1.2.4.1
MPNT_60122	*gltA*	Citrate synthase	2.3.3.16
MPNT_130021	*acnA*	Aconitate hydratase (aconitase)	4.2.1.3
MPNT_250013	*icd*	Isocitrate dehydrogenase	1.1.1.42
MPNT_290007	*sucA*	2-Oxoglutarate decarboxylase	1.2.4.2
MPNT_290006	*sucB*	2-Oxoglutarate dehydrogenase complex (dihydrolipoyltranssuccinase, E2 subunit)	2.3.1.61
MPNT_60044	*sucC*	Succinyl-CoA synthetase, beta subunit	6.2.1.5
MPNT_60045	*sucD*	Succinyl-CoA synthetase, alpha subunit	6.2.1.5
MPNT_50127	*sdhC*	Succinate dehydrogenase/fumarate reductase, cytochrome b subunit	1.3.5.1, 1.3.5.4
MPNT_50130	*sdhA*	Succinate dehydrogenase/fumarate reductase, flavoprotein subunit	1.3.5.1, 1.3.5.4
MPNT_50131	*sdhB*	Succinate dehydrogenase/fumarate reductase, iron-sulfur subunit	1.3.5.1, 1.3.5.4
MPNT_130050	*fumC*	Fumarate hydratase (Fumarase)	4.2.1.2
MPNT_10378	*mdh1*	Malate dehydrogenase	1.1.1.37
MPNT_10382	*mdh2*	Malate dehydrogenase	1.1.1.37

**Glycogen synthesis/degradation**

Identifier	Gene	Product	E.C. number
MPNT_50164	*glk*	Glucokinase	2.7.1.2
MPNT_120004	*pgcA*	Phosphoglucomutase	5.4.2.2
MPNT_30100	*glgA*	Glycogen synthase	2.4.1.21
MPNT_30011	*bglX*	Beta-glucosidase	3.2.1.21
MPNT_140031	*glgP1*	Glycogen phosphorylase	2.4.1.1
MPNT_30017	*glgP2*	Glycogen phosphorylase	2.4.1.1
**Respiratory chain**			
**Identifier**	**Gene**	**Product**	**E.C. number**
**Complex I**			
MPNT_40028	*nuoN*	NADH-quinone oxidoreductase subunit N	1.6.99.5
MPNT_40027	*nuoM*	NADH quinone-oxidoreductase subunit M	1.6.99.5
MPNT_40026	*nuoL*	NADH quinone-oxidoreductase subunit L	1.6.99.5
MPNT_40025	*nuoK*	NADH-quinone oxidoreductase subunit K	1.6.99.5
MPNT_40023	*nuoI*	NADH-quinone oxidoreductase subunit I	1.6.99.5
MPNT_110031	*nuoH*	NADH-quinone oxidoreductase subunit H	1.6.99.5
MPNT_110032	*nuoG*	NADH-quinone oxidoreductase subunit G	1.6.99.5
MPNT_110033	*nuoF*	NADH-quinone oxidoreductase subunit F	1.6.99.5
MPNT_110034	*nuoE*	NADH-quinone oxidoreductase subunit E	1.6.99.5
MPNT_110036	*nuoD*	NADH-quinone oxidoreductase subunit D	1.6.99.5
MPNT_110037	*nuoC*	NADH-quinone oxidoreductase subunit C	1.6.99.5
MPNT_110038	*nuoB*	NADH-quinone oxidoreductase subunit B	1.6.99.5
MPNT_30015	*nuoA*	NADH-quinone oxidoreductase subunit A	1.6.99.5
**Complex II**			
MPNT_50127	*sdhC*	Succinate dehydrogenase/fumarate reductase, cytochrome b subunit	1.3.5.1, 1.3.5.4

MPNT_50130	*sdhA*	Succinate dehydrogenase/fumarate reductase, flavoprotein subunit	1.3.5.1, 1.3.5.4
MPNT_50131	*sdhB*	Succinate dehydrogenase/fumarate reductase, iron-sulfur subunit	1.3.5.1, 1.3.5.4
**Alternative Complex III**			
MPNT_10279	*actA*	ACIII subunit A	
MPNT_10280	*actB*	ACIII subunit B	
MPNT_10281	*actC*	ACIII subunit C	
MPNT_10282	*actD*	ACIII subunit D	
MPNT_10283	*actE*	ACIII subunit E	
MPNT_10284	*actF*	ACIII subunit F	
MPNT_10285	*actG*	ACIII subunit G	
**Complex IV**			
MPNT_10081	*ctaB*	Protoheme IX farnesyltransferase	2.5.1.-
MPNT_10082	*ctaA*	Heme A synthase (cytochrome oxidase biogenesis protein CtaA)	1.3.-.-
MPNT_10286	*cyoC*	Cytochrome c oxidase subunit 3	1.9.3.1
MPNT_10290	*cyoA*	Cytochrome c oxidase subunit 2	1.9.3.1
MPNT_370004	*cyoB*	Cytochrome c oxidase subunit 1	1.9.3.1
MPNT_410012	*coxA*	Cytochrome c oxidase, subunit I	1.9.3.1
MPNT_110013	*cbaB*	Cytochrome c oxidase, subunit 2	1.9.3.1
MPNT_110014	*cbaA*	Cytochrome c oxidase, subunit 1	1.9.3.1
**Complex V**			
MPNT_10293	*atpC*	ATP synthase, F1 complex, epsilon subunit	3.6.3.14
MPNT_10294	*atpD*	ATP synthase, F1 complex, beta subunit	3.6.3.14
MPNT_10295	*atpG*	ATP synthase, F1 complex, gamma subunit	3.6.3.14
MPNT_10296	*atpA*	ATP synthase, F1 complex, alpha subunit	3.6.3.14

MPNT_10297	*atpH*	ATP synthase, F1 complex, delta subunit	3.6.3.14
MPNT_10298	*atpF*	ATP synthase, F0 sector, subunit b	3.6.3.14
MPNT_10299	*atpE*	ATP synthase, F0 sector, subunit c	3.6.3.14
MPNT_10300	*atpB*	ATP synthase, F0 sector, subunit a	3.6.3.14
**Nitrogen metabolism**			
**Identifier**	**Gene**	**Product**	**E.C. number**
MPNT_100073	*amtB 1*	Ammonium/ammonia transporter	
MPNT_250005	*amtB 2*	Ammonium/ammonia transporter	
MPNT_20071	*nrtC*	Nitrate ABC transporter, ATP binding protein	
MPNT_20072	*nrtA*	Nitrate ABC transporter, substrate-binding protein	
MPNT_20073	*nrtB*	Nitrate ABC transporter, permease protein	
MPNT_40047	*nrtD 1*	Nitrate ABC transporter, ATP-binding protein	
MPNT_40048	*nrtD 2*	Nitrate ABC transporter, ATP-binding protein	
MPNT_110017	*nasC*	Assimilatory nitrate reductase, catalytic subunit	1.7.-.-
MPNT_110016	*nasD*	Nitrite reductase	1.7.1.4
MPNT_50182	*cynS*	Cyanate hydratase	4.2.1.104
MPNT_410004	*norC*	Nitric oxide reductase, subunit C	1.7.2.5
MPNT_410005	*norB*	Nitric oxide reductase, subunit B	1.7.2.5
MPNT_80050	*glnA1*	Glutamine synthetase	6.3.1.2
MPNT_170017	*glnE*	Glutamine synthetase adenylyltransferase/adenylyl-removing enzyme (bifunctional)	2.7.7.42, 2.7.7.89
MPNT_210017	*glnA2*	Glutamine synthetase (fragment 1)	6.3.1.2
MPNT_210019	*glnA2*	Glutamine synthetase (fragment 2)	6.3.1.2
MPNT_40080	*gltB*	Glutamate synthase, large chain	1.4.1.13
**Sulfur metabolism**			

**Identifier**	**Gene**	**Product**	**E.C. number**
MPNT_580001	*cysP*	Sulfate ABC transporter, substrate binding protein	
MPNT_580002	*cysT*	Sulfate ABC transporter, permease protein	
MPNT_580003	*cysW*	Sulfate ABC transporter, permease subunit	
MPNT_580004	*cysA*	Sulfate ABC transporter, ATP-binding protein	
MPNT_10354	*cysD*	Sulfate adenylyltransferase subunit 2	2.7.7.4
MPNT_10355	*cysC*	Adenylyl-sulfate kinase	2.7.1.25
MPNT_20189	*sir1*	Sulfite reductase (Ferredoxin)	1.8.7.1
MPNT_20190	*cysH*	Phosphoadenosine phosphosulfate reductase	1.8.4.8
MPNT_60137	*sseA*	Sulfurtransferase	2.8.1.1
MPNT_10061	*cysJ1*	Sulfite reductase (NADPH) flavoprotein alpha- component	1.8.1.2
MPNT_40049	*cysJ2*	Sulfite reductase [NADPH] flavoprotein alpha- component	1.8.1.2
MPNT_10197	*cysE*	Serine acetyltransferase	2.3.1.30
MPNT_110064	*cysK*	Cysteine synthase	2.5.1.47
MPNT_180031	*mtoX*	Methanethiol oxidase	
**Phosphate metabolism**			
**Identifier**	**Gene**	**Product**	**E.C. number**
MPNT_50133	*pstB*	Phosphate ABC transporter, ATP-binding protein	3.6.3.27
MPNT_50134	*pstA*	Phosphate ABC transporter, permease protein	
MPNT_50135	*pstC*	Phosphate ABC transporter, permease protein	
MPNT_50136	*pstS*	Phosphate ABC transporter, phosphate-binding protein	
